# Supplementary material for: Hearing Aids Reduce Daily-Life Fatigue and Increase Social Activity: A Longitudinal Study
Source: Trends Hear. 2021 Nov 8;25:23312165211052786. doi: 10.1177/23312165211052786 (PMC8579337; doi:10.1177/23312165211052786)
Supplement: sj-docx-2-tia-10.1177_23312165211052786 - Supplemental material for Hearing Aids Reduce Daily-Life Fatigue and Increase Social Activity: A Longitudinal Study [file sj-docx-2-tia-10.1177_23312165211052786.docx]

**Supplementary digital content 2**


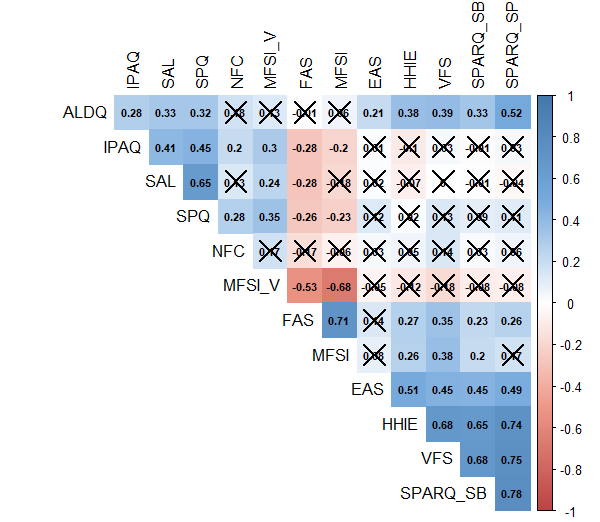
Figure: Correlation matrix of baseline questionnaires

Figure: Correlation matrix of baseline questionnaires. Spearman rank correlation coefficients are colour coded and labelled. Where there was no significant correlation (p > .05), the square is crossed. MFSI_V = MFSI vigour subscale; SPARQ_SB = SPARQ social behaviours subscale; SPARQ_SP = SPARQ social perceptions subscale. Other questionnaire abbreviations can be found in the outcome measures section of the main manuscript.


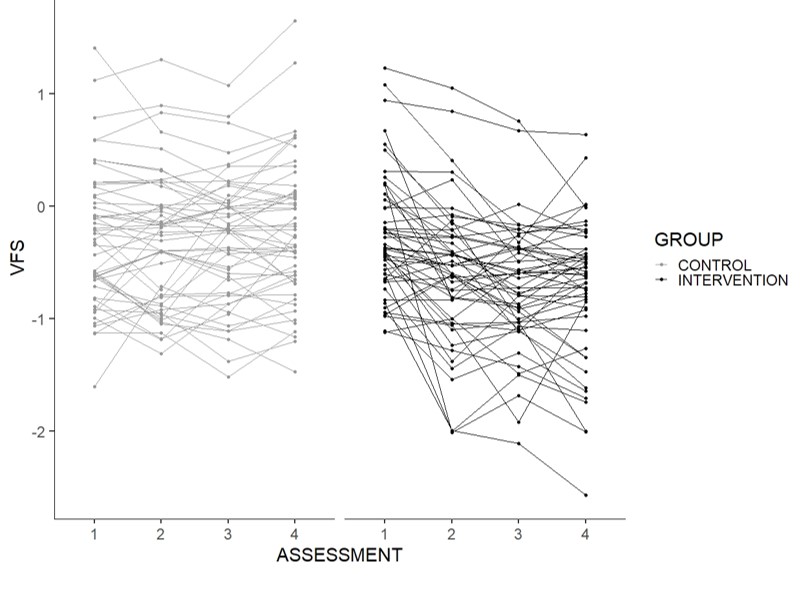
Figure: Individual VFS-AHL score trajectories over time by group

Figure: Individual VFS-A-40 score trajectories over time by group. Each line represents one participant’s VFS-A-40 score from baseline to session four. VFS = 40-item Vanderbilt Fatigue Scale for Adults
